# Statistics of 250 Cases of Cancer of the Breast

**Published:** 1880-09

**Authors:** 


### STATISTICS OF 250 CASES OF CANCER OF THE BREAST.

Dr. Oldekop publishes an extended report of 250 cases of carcinoma of the mamma, which were treated in Prof. Esmarch's wards in Kiel, between the years 1850 and 1878. Of these cases 21 were not operated on. Of the remaining 229, 23 died in consequence of the operation; in 109 the tumors returned; in 43 the tumors did not recur, some of these patients being still alive, while others have died of intercurrent diseases; in 54 the patients were lost sight of after they left the hospital. The majority of the patients were between 46 and 50 years of age; the average age at which the disease first made its appearance was 48.4 years. Of the patients, 208 were married, and 30 single. Of 103 who had borne children, 36 had suffered from puerperal mastitis. In 9 cases the tumor developed from nodules left by previous mastitis. The statements with regard to previous injury were uncertain. The cancer affected the right breast in 123 cases, and the left in 102. The upper and outer half of the gland was most frequently affected. In 11 cases hereditary predisposition existed, and in 60 cases it could be positively excluded. In 31 cases in which the axillary glands were not involved, the average duration of life after the operation was 45.1 months; period of freedom from relapse, 6 months. In 57 cases in which the glands were involved, the average duration of life after the operation was 34.8 months; period of freedom from relapse, 2.5 months. The average duration of life from the first appearance of the disease was, in the cases not operated on, 22.6 months, and in the cases operated on, 38.1 months. On 225 patients, 287 operations were performed, with 23 deaths. Out of 184 operations performed before the introduction of Lister's method, there were 16 deaths, a mortality of 8.7 per cent.; out of 77 performed under antiseptic precautions, there were 7 deaths, a mortality of 9.1 per cent. The average period of convalescence was formerly 5.2 weeks, but after the adoption of Lister's method it fell to 4.6 weeks. In 40.9 per cent. of the patients the entire mamma with the glands was removed (mortality, 13 per cent.) Of the 23 deaths from the operation, 12 were due to accidental surgical diseases, 4 to collapse and secondary hemorrhage, 1 to pneumonia, and 6 to causes that could not be clearly ascertained. Erysipelas occurred 15 times, and proved fatal in 5 cases. In 46.4 per cent. of the cases the recurrent tumors appeared within the first three months after the operation; after that period the recurrences diminished steadily in frequency, and after one year they only occurred in 18 cases, or 16 per cent. A reappearance of the tumor after three years' interval was only observed in one case, and in that there was some room for doubt. Hence three years may be regarded as the limit for the appearance of recurrent tumors. If this be accepted as correct, 23 of Esmarch's cases may be regarded as definitely cured. The seat of recurrence was far more frequently the cicatrix than the axilla; and when the operation was limited to the removal of axillary glands, the recurrent tumors generally appeared in the axilla. Dr. Oldekop concludes his paper with brief synopses of the histories of the 250 cases.—*Langenbeck's Archiv.*, vol. 24.

